# Enhanced Autonomous Vehicle Positioning Using a Loosely Coupled INS/GNSS-Based Invariant-EKF Integration

**DOI:** 10.3390/s23136097

**Published:** 2023-07-02

**Authors:** Ahmed Ibrahim, Ashraf Abosekeen, Ahmed Azouz, Aboelmagd Noureldin

**Affiliations:** 1 Electrical Engineering Branch, Military Technical College (MTC), Cairo 11766, Egypt; 2 Electrical and Computer Engineering, Royal Military College of Canada (RMCC), Kingston, ON K7K 7B4, Canada

**Keywords:** INS, MEMS-IMU, GNSS, INS/GNSS integration, EKF, IEKF, positioning, navigation

## Abstract

High-precision navigation solutions are a main requirement for autonomous vehicle (AV) applications. Global navigation satellite systems (GNSSs) are the prime source of navigation information for such applications. However, some places such as tunnels, underpasses, inside parking garages, and urban high-rise buildings suffer from GNSS signal degradation or unavailability. Therefore, another system is required to provide a continuous navigation solution, such as the inertial navigation system (INS). The vehicle’s onboard inertial measuring unit (IMU) is the main INS input measurement source. However, the INS solution drifts over time due to IMU-associated errors and the mechanization process itself. Therefore, INS/GNSS integration is the proper solution for both systems’ drawbacks. Traditionally, a linearized Kalman filter (LKF) such as the extended Kalman filter (EKF) is utilized as a navigation filter. The EKF deals only with the linearized errors and suppresses the higher orders using the Taylor expansion up to the first order. This paper introduces a loosely coupled INS/GNSS integration scheme using the invariant extended Kalman filter (IEKF). The IEKF state estimate is independent of the Jacobians that are derived in the EKF; instead, it uses the matrix Lie group. The proposed INS/GNSS integration using IEKF is applied to a real road trajectory for performance validation. The results show a significant enhancement when using the proposed system compared to the traditional INS/GNSS integrated system that uses EKF in both GNSS signal presence and blockage cases. The overall trajectory 2D-position RMS error reduced from 19.4 m to 3.3 m with 82.98% improvement and the 2D-position max error reduced from 73.9 m to 14.2 m with 80.78% improvement.

## 1. Introduction

The main challenge in autonomous vehicle (AV) navigation is providing an accurate, reliable, and continuous navigation solution in all environments. Therefore, the most common source of navigation solutions for AVs is the GNSS because of its long-term solution stability [1]. However, there are several situations where the GNSS cannot provide either an accurate solution or any solution at all. Particularly, in urban areas with tunnels, underground parking, and high-rise buildings where a line of sight ‘LOS’ between at least four GNSS satellites and the receiver’s antenna is lost, a degraded or complete solution loss occurs [2]. Therefore, there is an important need for a backup system that is capable of providing an uninterrupted navigation solution, such as the inertial navigation system (INS). The INS solution is immune to signal interference but its quality depends mainly on the inertial measuring unit (IMU) grade [3].

However, IMUs are vulnerable to various error sources which can be categorized into deterministic and stochastic errors. The deterministic errors are mitigated by calibration while the stochastic errors cannot be calibrated but they can be modeled. Despite that, the INS solution drifts over time because its mechanization utilizes Newton’s laws of motion to provide position, velocity, and attitude (PVA). However, for low-cost IMUs there are unbounded errors affecting its raw measurements that derive the INS mechanization [4,5].

Therefore, neither GNSS nor INS satisfies the AV navigation requirements. Thus, an integration between the two systems is applied to benefit from each system’s advantages and decrease each system’s disadvantages.

Classically, an extended Kalman filter (EKF) has been utilized for INS/GNSS integration. Moreover, the EKF deals only with the linearized errors and suppresses the higher orders using the Taylor expansion up to the first order [1,6]. Consequently, the dynamic system model using the EKF is a linearized system that denies the nonlinear terms of its associated noise. Thus, using EKF provides unbounded errors in the updated error covariance which leads to solution divergence [7,8].

Unlike the EKF, the unscented Kalman filter (UKF) utilizes the higher-order terms of the system’s noise. Therefore, the UKF provides a more realistic approximation of the system’s dynamic model [9]. In particular, the UKF uses a sampling technique to overcome the EKF limitations by setting sets of sampling points in the normal random distribution of the system’s states [10,11]. Furthermore, the chosen sampling points capture almost the right means and covariance of the system’s noise. Thereafter, the updated mean and covariance are precisely propagated to the second order of the nonlinear dynamic system model. Thus, the UKF provides a better estimation, unlike the EKF which only uses a first order approximation. However, the UKF process time and computational cost are large compared to the EKF [12,13,14].

Hence, the researchers inserted machine learning (ML) techniques into the navigation algorithm, in which the navigation parameters are estimated, predicted, and classified. Moreover, these methods are used to simplify the selection of appropriate sensors in a plug-and-play manner as an alternative to the Kalman filter. This causes the integration procedure and raw measurements to be randomly chosen. Hence, developing a robust predictive model for the INS errors during GNSS outages is made possible by training the ML model. Moreover, it can be used to enhance visual positioning and reduce the consequences of multipath effects, spoofing, and jamming. Unfortunately, the process of training takes a long time and has high processing costs [15].

Generally, estimators have to get the state estimation error to converge to a minimum in any filter-based sensor fusion system. Therefore, various navigation filters are utilized in the integration process such as UKF and particle filter (PF), but its integration is more complex than the traditional EKF [16]. Furthermore, artificial intelligence (AI) techniques, such as conventional neural networks (CNNs), fuzzy inference systems (FISs), adaptive neuro-fuzzy inference systems (ANFISs), and fuzzy clustering techniques, play an important role in providing error estimation for the integrated INS/GNSS systems [17,18,19,20,21]. Consequently, an accurate, reliable, and continuous navigation solution is achieved. Unfortunately, the process of training takes a long time and has high processing costs [22].

The invariant extended Kalman filter (IEKF) is based on the symmetry-preserving observer theory which claims that the estimation error is invariant under the action of a Lie group matrix [23,24,25,26]. The main advantage of IEKF over EKF is that the state linearization and measurement models are independent of the current estimation of the state leading to state-independent Jacobians at any linearization point [27,28]. Moreover, the EKF uses the Taylor expansion form but the IEKF uses the Riccati equation for error estimation [29,30]. On the other hand, the IEKF methodology provides a number of distinctively advantageous properties such as symmetry-preserving observer, reliable geometrical models for quaternion estimation, improved accuracy, consistency in the estimation of the state covariance matrix, numerical stability, handling of nonlinearities, handling of sensor biases and handling of kinematic constraints, reduced sensitivity to initialization errors, and a significant predicted convergence domain compared to the typical EKF-based techniques [31,32].

This paper introduces the IEKF as a navigation filter using quaternion rotation for the INS/GNSS integration scheme in which its observable state variables are made to converge within an area of attraction. Moreover, it is independent of the system’s trajectory and relies on the IMU dynamics. In addition, this paper shows how the specified system can be used as a process model for the IEKF as it satisfies the property of log-linear error dynamics [33,34]. The contributions of this paper are summarized as follows:

- The IEKF-based quaternion rotation is applied using real road data from global positioning system (GPS) receiver measurements and low-cost IMU in a loosely coupled integration scheme.
- A performance comparison between the traditional EKF and the proposed IEKF is presented including GPS outage.
- An analysis of the two filters’ performance during various dynamics with several average speeds and traveled distances is illustrated for validation purposes.

The organization of the paper’s remaining sections is as follows: Section 2 provides the methodology of using IEKF as a navigation filter of loosely coupled INS/GPS integration. Section 3 provides the experimental setup and the specifications of the sensors utilized in the road trajectory. Section 4 describes the experimental setup and results. Finally, Section 5 concludes the proposed methodology and gives recommendations for future work.

## 2. Methodology

### 2.1. Inertial Navigation Systems (INS)

The strap-down inertial navigation system (INS) depends on the IMU measurements, the navigation mechanization, and the initial states from the GPS or manually entered by the user. Basically, the IMU utilizes a coincident 3 accelerometers, to provide the measured specific forces, and 3 gyroscopes to provide the measured angular rates [35,36,37,38].

The INS as a self-contained navigation system uses the knowledge of its carrying platform’s initial states (position, velocity, and attitude (P,V,A)) and accordingly updates its current states.

Starting with the angular rates ($\omega_{x},\omega_{y},\omega_{z}$), the attitude angles pitch, roll, and yaw (p, r , y) can be obtained after calculating the transformation matrix. Simultaneously, the rotation matrix is applied to the accelerations $\left(f_{x},\;f_{y},\;f_{z}\right)$ to convert them into forces in the navigation/ local-level frame (LLF). Afterward, the velocity is provided by integrating the transformed forces and the position is derived by integrating the calculated velocity [1,39]. Finally, the produced PVA becomes the initial state of the next epoch. The attitude in the quaternion is represented in Equation (1).

(1) $$A=\left[\begin{array}{c} q_{0}\\ q_{1}\\ q_{2}\\ q_{3} \end{array}\right]$$

where *A* is the attitude and $\left(q_{0},q_{1},q_{2},q_{3}\right)$ are the rotation matrix’s quaternion parameters.

The attitude rates $\dot{A}$ are calculated as Equation (2).

(2) $$\dot{A}=\left[\begin{array}{c} \dot{q_{0}}\\ \dot{q_{1}}\\ \dot{q_{2}}\\ \dot{q_{3}} \end{array}\right]=0.5\left[\begin{array}{cccc} 0 & \omega_{x} & -\omega_{y} & -\omega_{z}\\ \omega_{x} & 0 & \omega_{z} & -\omega_{y}\\ \omega_{y} & -\omega_{z} & 0 & \omega_{x}\\ \omega_{z} & \omega_{y} & -\omega_{x} & 0 \end{array}\right]\left[\begin{array}{c} q_{0}\\ q_{1}\\ q_{2}\\ q_{3} \end{array}\right]$$

Also, the quaternion attitude can be transferred to Euler’s angles roll, pitch, and yaw, respectively, as in Equation (3).

(3) $$\left[\begin{array}{c} \phi \\ \theta \\ \psi \end{array}\right]=\left[\begin{array}{c} \left.atan2\left(2\left.\left.q_{2}q_{3}\right.+\left.{2q}_{1}q_{0}\right.\right.),\left(q_{3}^{2}\right.+\left.q_{0}^{2}\right.-\left.q_{1}^{2}\right.-\left.q_{2}^{2}\right)\right)\right.\\ -asin\left(2\left.\left.q_{1}q_{3}\right.-\left.{2q}_{2}q_{0}\right.\right.\right)\\ atan2\left(\left(2\left.\left.q_{1}q_{2}\right.+\left.{2q}_{0}q_{3}\right.\right.\right),\left(q_{0}^{2}\right.+\left.q_{1}^{2}\right.-\left.q_{2}^{2}\right.-\left.q_{3}^{2}\right)\right) \end{array}\right]$$

where ϕ is the roll angle in radians, θ is the pitch angle in radians, and ψ is the yaw angle in radians.

The velocity (v) components in the LLF are shown in Equation (4). They are derived from the transformed forces as in Equations (7) and (8).

(4) $$v=\left[\begin{array}{c} V_{N}\\ V_{E}\\ V_{D} \end{array}\right]$$

where $V_{N}$ is the north velocity, $V_{E}$ is the east velocity, and $V_{D}$ is the down velocity.

The position (p) components are shown in Equation (5)

(5) $$p=\left[\begin{array}{c} \varphi \\ \lambda \;\\ h \end{array}\right]$$

where φ is the latitude, λ is the longitude, and h is the altitude.

Equation (6) shows the transformation matrix from the body frame to LLF using quaternion states [40].

(6) $$C_{b}^{n}=\left[\begin{array}{ccc} q_{1}^{2}+q_{0}^{2}-q_{2}^{2}-q_{3}^{2} & 2(q_{1}q_{2}-q_{3}q_{0}) & 2(q_{2}q_{3}+q_{2}q_{0})\\ 2(q_{1}q_{2}+q_{3}q_{0}) & q_{2}^{2}+q_{0}^{2}-q_{1}^{2}-q_{3}^{2} & 2(q_{2}q_{3}-q_{1}q_{0})\\ 2(q_{1}q_{3}-q_{2}q_{0}) & 2(q_{2}q_{3}+q_{1}q_{0}) & q_{3}^{2}+q_{0}^{2}-q_{1}^{2}-q_{2}^{2} \end{array}\right]$$

The specific forces can be transformed into LLF using the transformation matrix $C_{b}^{n}$ and are obtained as in Equation (7).

(7) $$\left[\begin{array}{c} F_{N}\\ F_{E}\\ F_{D} \end{array}\right]=\;C_{b}^{n}\left[\begin{array}{c} f_{x}\\ f_{y}\\ f_{z} \end{array}\right]$$

The velocity rates can be obtained as in Equation (8).

(8) $$\left[\begin{array}{c} {\dot{V}}_{N}\\ {\dot{V}}_{E}\\ {\dot{V}}_{D} \end{array}\right]=\left[\begin{array}{ccccccc} 1 & 0 & 0 & 0 & -\left(\dot{\lambda }+2w_{e}sin\left(\varphi \right)\right) & \dot{\varphi } & 0\\ 0 & 1 & 0 & \left(\dot{\lambda }+2w_{e}sin\left(\varphi \right)\right) & 0 & \left(\dot{\lambda }+2w_{e}cos\left(\varphi \right)\right) & 0\\ 0 & 0 & 1 & -\dot{\varphi } & -\left(\dot{\lambda }+2w_{e}cos\left(\varphi \right)\right) & 0 & 1 \end{array}\right]\left[\begin{array}{c} F_{N}\\ F_{E}\\ F_{D}\\ V_{N}\\ V_{E}\\ V_{D}\\ g \end{array}\right]$$

where $\dot{\lambda }$ is the longitude rate, $\dot{\varphi }$ is the latitude rate, $w_{e}=7.2921158\times 10^{-5}$ rad/s is the Earth’s rotation rate, and *g* is the modeled gravity that is calculated as shown in Equation (9).

(9) $$g=\;g_{WGS0}\frac{1+g_{WGS1}sin\left(\varphi \right)}{{\left[1-\;E^{2}{sin}^{2}\left(\varphi \right)\right]}^{\frac{1}{2}}}-\left\lceil 3.0877\times 10^{-6}-0.0044\times 10^{-6}{sin}^{2}\left(\varphi \right)\right\rceil h+0.072\times 10^{-12}$$

where $g_{WGS0}$= 9.78032677 m/s${}^{2}$ is the nominal gravity measured at the equator, $g_{WGS1}$= 0.00193185138639 m/s${}^{2}$ is the gravity formula constant, and $E^{2}=0.0818191908426$ is the 2nd eccentricity that identifies Earth’s flattening.

The position rates (velocity components) are calculated as shown in Equation (10) [1,41].

(10) $$\left[\begin{array}{c} \dot{\varphi }\\ \dot{\lambda }\\ \dot{h} \end{array}\right]\;=\;\left[\begin{array}{c} \frac{V_{N}}{R_{M}+h}\;\\ \frac{V_{E}}{\left(R_{N}+h\right)cos\left(\varphi \right)}\;\\ {-V}_{D} \end{array}\right]$$

where $R_{M}$ and $R_{N}$ are semi-major and semi-minor axes radii of Earth’s ellipsoid model, respectively.

Nevertheless, the INS provided navigation solution suffers from error growth over time as a result of the target’s acceleration two-times integration process. The errors in the INS can be classified as deterministic or stochastic/random. Deterministic errors include bias offset, scale factor, and axis misalignment. In contrast, random errors include bias drift, bias stability, scale factor stability, and noise. Deterministic errors can be reduced or compensated if the sensors, particularly high-end sensors, are properly calibrated, whereas stochastic errors are modeled randomly to reduce their effect [1,42].

Moreover, the categorization of the INS systems relies mainly on both the accuracy and error reduction capabilities of the utilized IMU. Therefore, the need to compensate for the low-cost commercial IMUs’ errors has been elevated by assisting the INS system with other systems that have long-term stability, such as the GNSS systems. The integration between the two systems produces a more accurate and reliable navigation system. However, the error growth rate between each consecutive update is limited by the quality of the INS system only. Traditionally, EKF is utilized in open- or closed-loop schemes to bind the error growth. Unfortunately, the EKF is bounded by the first-order part of the system’s errors derived by using Taylor expansion as an initial step.

**Figure 1:**
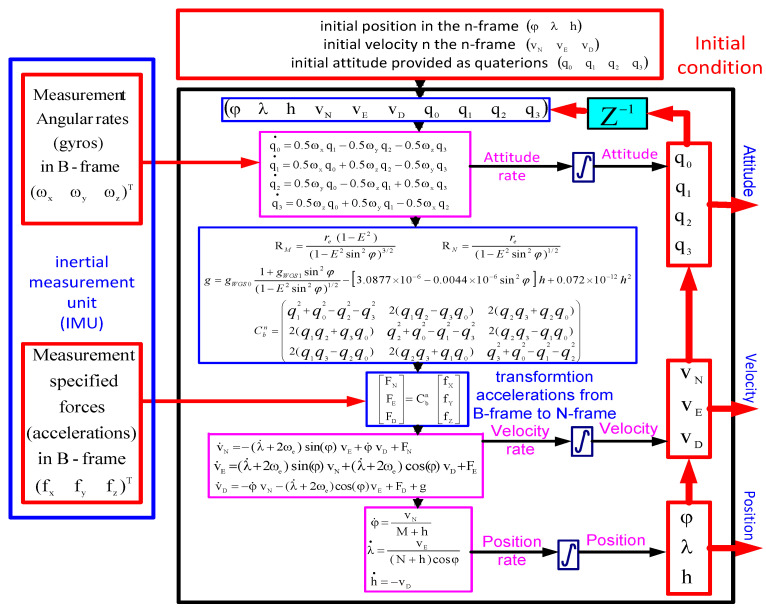
A detailed strap-down INS mechanization block diagram.

A detailed block diagram of the utilized INS system mechanization using the quaternion angles is illustrated in Figure 1.

### 2.2. Extended Kalman Filter

The EKF is applied to be a closed-loop configuration, in which the state’s error can be estimated and fed back again to the INS sensor to obtain more accurate INS solutions and maintain the linearity of the system model [6]. Moreover, the EKF uses the Taylor expansion as a linearizing technique for nonlinear systems and the EKF deals only with the linearized errors and suppresses the higher orders using the Taylor expansion up to the first order. Thus, using EKF provides unbounded errors in the updated error covariance which leads to solution divergence [1,43].

#### 2.2.1. State Representation

To estimate (p,v,A) of the vehicle in the navigation frame, the continuous system model states are represented by Equation (11).

(11) $$\delta \dot{x}=F\delta x+Gw$$

The state vector including error components is given by Equation (12).

(12) $$\delta x_{9\times 1}^{e}={\left[\delta p_{3\times 1}^{e},\delta v_{3\times 1}^{e},\delta A_{3\times 1}^{e}\right]}^{T}$$

where $\delta p^{e}={\left[\delta \varphi ,\delta \lambda ,\delta h\right]}^{T}$ are the position error states, $\delta v^{e}={\left[\delta v_{N},\delta v_{E},\delta v_{D}\right]}^{T}$ are the velocity error states, and $\delta A^{e}={\left[\delta p,\delta r,\delta y\right]}^{T}$ are the attitude error states.

Furthermore, *F* is the dynamic coefficient matrix and can described as in Equation (13).

(13) $$F=\left[\begin{array}{ccc} 0_{3\times 3} & F_{p} & 0_{3\times 3}\\ 0_{3\times 3} & 0_{3\times 3} & F_{v}\\ 0_{3\times 3} & F_{A} & 0_{3\times 3} \end{array}\right]$$

where $F_{p}$ denotes the position’s coefficient matrix, $F_{v}$ represents the velocity’s coefficient matrix, and $F_{A}$ denotes the attitude’s coefficient matrix, and each matrix dimension is 3×3 as detailed in [1].

Finally, *G* denotes the noise distribution, represented by Equation (14), and *w* is the unit-variance white Gaussian noise.

(14) $$G=\left[\begin{array}{c} \sigma_{p3\times 1}\\ \sigma_{v3\times 1}\\ \sigma_{A3\times 1} \end{array}\right]$$

The system model for a loosely coupled integration is given by Equation (15).

(15) $$\left[\begin{array}{c} \delta {\dot{p}}_{3\times 1}^{e}\\ \delta {\dot{v}}_{3\times 1}^{e}\\ {\dot{A}}_{3\times 1}^{e} \end{array}\right]=\left[\begin{array}{ccc} 0_{3\times 3} & F_{p} & 0_{3\times 3}\\ 0_{3\times 3} & 0_{3\times 3} & F_{v}\\ 0_{3\times 3} & F_{A} & 0_{3\times 3} \end{array}\right]\left[\begin{array}{c} \delta p_{3\times 1}^{e}\\ \delta v_{3\times 1}^{e}\\ A_{3\times 1}^{e} \end{array}\right]+\left[\begin{array}{c} \sigma_{p3\times 1}\\ \sigma_{v3\times 1}\\ \sigma_{A3\times 1} \end{array}\right]\;w$$

This system model equation can be discretized as in Equation (16).

(16) $$\delta x_{n}=\left(I+F\Delta t\right)\delta x_{n-1}+G\Delta w_{n-1}$$

#### 2.2.2. Measurement Model

The measurement model in the discrete-time domain is given by Equation (17).

(17) $$\delta z_{n}=H_{n}\delta x_{n}+\eta_{n}$$

(18) $$\delta z_{n}=\left[\begin{array}{c} p_{INS}^{e}-p_{GPS}^{e} \end{array}\right]=\left[\begin{array}{c} \varphi_{INS}-\varphi_{GPS}\\ \lambda_{INS}-\lambda_{GPS}\\ h_{\mathrm{INS}\;}-h_{GPS} \end{array}\right]$$

where $\delta x_{n}$ is the state vector, $\eta_{n}$ is a vector of measurement noise, and $H_{n}$ is the measurement design matrix at time $t_{n}$.

$H_{n}$ in a simple form is given in Equation (19).

(19) $$H_{n}=\left[\begin{array}{cc} I_{3\times 3} & 0_{3\times 6} \end{array}\right]$$

The full measurement model is given as in Equation (20).

(20) $$\left[\begin{array}{c} p_{INS}^{e}-p_{GPS}^{e} \end{array}\right]=\left[\begin{array}{cc} I_{3\times 3} & 0_{3\times 6} \end{array}\right]\left[\begin{array}{c} \delta x_{n} \end{array}\right]+\left[\begin{array}{c} \eta_{p} \end{array}\right]$$

The expanded form of the measurement model is given as in Equation (21).

(21) $${\left[\begin{array}{c} \varphi_{INS}-\varphi_{GPS}\\ \lambda_{INS}-\lambda_{GPS}\\ h_{INS}-h_{GPS} \end{array}\right]}_{n}={\left[\begin{array}{ccccccccc} 1 & 0 & 0 & 0 & 0 & 0 & 0 & 0 & 0\\ 0 & 1 & 0 & 0 & 0 & 0 & 0 & 0 & 0\\ 0 & 0 & 1 & 0 & 0 & 0 & 0 & 0 & 0 \end{array}\right]}_{n}\left[\begin{array}{c} \delta p_{3\times 1}^{e}\\ \delta v_{3\times 1}^{e}\\ A_{3\times 1}^{e} \end{array}\right]+{\left[\begin{array}{c} \eta_{\varphi }\\ \eta_{\lambda }\\ \eta_{h} \end{array}\right]}_{n}$$

The covariance matrix of the measurement model error $R_{n}$ which contains the variances of the measured states is given as in Equation (22).

(22) $$R_{n}=\left[\begin{array}{ccc} \sigma_{\varphi }^{2} & 0 & 0\\ 0 & \sigma_{\lambda }^{2} & 0\\ 0 & 0 & \sigma_{h}^{2} \end{array}\right]$$

The covariance matrix of the states prediction $P_{n}$ which contains the variances of predicted states is given as in Equation (23). Moreover, it is a 9×9 element square matrix.

(23) $$P_{n}=\left[\begin{array}{ccc} \sigma_{p_{3\times 3}}^{2} & 0_{3\times 3} & 0_{3\times 3}\\ 0_{3\times 3} & \sigma_{V_{3\times 3}}^{2} & 0_{3\times 3}\\ 0_{3\times 3} & 0_{3\times 3} & \sigma_{A_{3\times 3}}^{2} \end{array}\right]$$

### 2.3. Invariant Extended Kalman Filter (IEKF)

The states of IEKF are not the typical Jacobian linearization along a trajectory as in the traditional EKF. On the contrary, the invariant error on the Lie group can be exactly recovered from its solution using the Riccati equation [44,45,46,47,48]. In this section, the IEKF utilizes position, velocity, and attitude (PVA) from the INS solution and the position from the GPS receiver as its control input parameters in a loosely coupled scheme.

Let a matrix Lie group denoted by G be the group of 3×3 rotation matrices that preserve orientation and its Lie algebra is the space of skew-symmetry matrices denoted by g that takes an element in the tangent space of G at the identity to its corresponding matrix representation as follows [46]:

(24) $$L_{g}:R^{\mathrm{dimg}\;}\to g$$

then the exponential map is shown as in the equation that relates a matrix Lie group to its associated Lie algebra as follows:

(25) $$\exp:R^{\mathrm{dimg}}\to G$$

and

(26) $$\exp\left(\xi \right)=\exp_{m}\left(L_{g}\left(\xi \right)\right)$$

where $\exp_{m}\left(\;\cdot \;\right)$ is the standard matrix exponential.

A process dynamics evolving on the Lie group is shown as follows:

(27) $$\frac{d}{dt}X_{t}=f_{u_{t}}\left(X_{t}\right)$$

where the state $X_{t}$ belongs to the Lie group G and $u_{t}$ is an input variable as shown in Equation (29), the true state trajectory is $X_{t}$, and the estimated trajectory of it is ${\bar{X}}_{t}$; then, the state estimation error is defined using left multiplication of $X_{t}^{-1}$ as shown in Equation (28).

(28) $$\begin{array}{c} \eta_{t}^{l}=X_{t}^{-1}{\bar{X}}_{t}={\left(\Gamma {\bar{X}}_{t}\right)}^{-1}\left(\Gamma X_{t}\right) \end{array}$$

where $\eta_{t}^{l}$ is the left-invariant errors between two trajectories $X_{t}$ and ${\bar{X}}_{t}$ and Γ∈G is an arbitrary element of the group, and

(29) $$u_{t}=\left[\begin{array}{c} \omega_{t}\\ a_{t} \end{array}\right]$$

A system is an affine group if the system dynamics $f_{u_{t}}\left(\;\cdot \;\right)$ satisfies that:

(30) $$f_{u_{t}}\left(X_{1}X_{2}\right)=f_{u_{t}}\left(X_{1}\right)X_{2}+X_{1}f_{u_{t}}\left(X_{2}\right)-X_{1}f_{u_{t}}\left(I_{d}\right)X_{2}$$

for all t>0 and $X_{1},X_{2}\in G$

Where $I_{d}$ represents the identity matrix. Moreover, if this condition is satisfied then the left-invariant error dynamics are trajectory independent and satisfy Equation (31).

(31) $$\frac{d}{dt}\eta_{t}^{l}=g_{u_{t}}^{l}\left(\eta_{t}^{l}\right)$$

where

(32) $$g_{u_{t}}^{l}\left(\eta \right)=f_{u_{t}}\left(\eta \right)-f_{u_{t}}\left(I_{d}\right)\eta$$

#### 2.3.1. State Representation

To estimate $\left(p_{t},v_{t},A_{t}\right)$ of the vehicle in the navigation frame, the continuous system model states are represented by Equation (24).

(33) $$X_{t}=\left[\begin{array}{ccc} p_{t_{3\times 1}} & v_{t_{3\times 1}} & C_{t_{3\times 3}}\\ 0 & 1 & 0_{1\times 3}\\ 1 & 0 & 0_{1\times 3} \end{array}\right]$$

where the system model is denoted by $X_{t}$, the position $p_{t}$ states (latitude, longitude, and altitude(φ,λ,h)), the velocity $v_{t}$ states ($V_{N},V_{E},V_{D}$), the transformation from the body frame to the navigational frame $C_{t}$ states, and the index t refers to the time invariant of IEKF at time (t).

Moreover, the adjoint operator $Ad_{X_{t}}$ plays a key role in the theory of Lie groups, it is as described in the adjoint map, and it represents the linear map of the Lie group as follows [46]:

(34) $${\mathrm{Ad}}_{X_{t}}\left(L_{g}\left(\xi \right)\right)=X_{t}L_{g}\left(\xi \right)X_{t}^{-1}$$

where ξ is the invariant error, $L_{g}\left(\xi \right)$ is the log of the invariant error, and X is the state vector.

(35) $$L_{g}\left(\xi \right)=L_{g}\left(\left[\begin{array}{c} \xi^{p_{t}}\\ \xi^{v_{t}}\\ \xi^{C_{t}} \end{array}\right]\right)={\left[\begin{array}{ccc} \xi_{3\times 1}^{p_{t}} & \xi_{3\times 1}^{v_{t}} & \xi_{3\times 3}^{C_{t}}\\ 0 & 0 & 0_{1\times 3}\\ 0 & 0 & 0_{1\times 3} \end{array}\right]}_{5\times 5}$$

where $\xi_{3\times 1}^{p_{t}}$ is the position invariant error, $\xi_{3\times 1}^{v_{t}}$ is the velocity invariant error, and $\xi_{3\times 3}^{C_{t}}$ the transformation matrix.

The exponential mapping is given as in Equation (36).

(36) $$\exp\left(\xi \right)=I_{5}+S+\frac{1-\cos∥\xi ∥}{{∥\xi ∥}^{2}}S^{2}+\frac{∥\xi ∥-\sin∥\xi ∥}{{∥\xi ∥}^{3}}S^{3}$$

where $S=L_{g}\left(\xi \right)$.

The adjoint operator matrix is given as shown in Equation (37).

(37) $${\mathrm{Ad}}_{X_{t}}=\left[\begin{array}{ccc} P_{t} & 0 & C_{t}\\ v_{t} & C_{t} & 0\\ C_{t} & 0 & 0 \end{array}\right]$$

The dynamic system model can be expressed as follows:

(38) $$\begin{array}{cc} \frac{d}{dt}X_{t} & =\left[\begin{array}{ccc} v_{t} & C_{t}{\tilde{a}}_{t}+g & C_{t}{\tilde{\omega }}_{t}^{\wedge }\\ 0 & 0 & 0_{1\times 3}\\ 0 & 0 & 0_{1\times 3} \end{array}\right]-\left[\begin{array}{ccc} p_{t} & v_{t} & C_{t}\\ 0 & 0 & 0_{1\times 3}\\ 0 & 0 & 0_{1\times 3} \end{array}\right]\left[\begin{array}{ccc} 0_{3\times 1} & w_{t}^{f} & {\left(w_{t}^{g}\right)}^{\wedge }\\ 0 & 0 & 0_{1\times 3}\\ 0 & 0 & 0_{1\times 3} \end{array}\right]\\ \; & =f_{u_{t}}\left(X_{t}\right)-X_{t}L_{g}\left(w_{t}\right) \end{array}$$

where $w_{t}={\left[{\left(w_{t}^{g}\right)}^{T},{\left(w_{t}^{f}\right)}^{T},0_{1\times 3}\right]}^{T}$, ${\left(\;\cdot \;\right)}^{\wedge }$ denotes a 3×3 skew-symmetric matrix, $w_{t}^{g}$ is the noise due to the IMUs’ gyroscopes, and $w_{t}^{f}$ is the noise due to the IMUs’ accelerometers. Moreover, depending on Equation (38), then the system dynamics [$f_{u_{t}}\left(\;\cdot \;\right)$] satisfy the affine group property as mentioned in Equation (30). Therefore, the left-invariant error dynamics will evolve independently of the system’s state and are represented as in Equation (39).

(39) $$\begin{array}{cc} \frac{d}{dt}\eta_{t}^{l} & =f_{u_{t}}\left(\eta_{t}^{l}\right)-f_{u_{t}}\left(I_{d}\right)\eta_{t}^{l}+L_{g}\left(w_{t}\right)\eta_{t}^{l}\\ \; & =g_{u_{t}}^{l}\left(\eta_{t}^{l}\right)+L_{g}\left(w_{t}\right)\eta_{t}^{l} \end{array}$$

Moreover, the invariant error satisfies a log-linear property if $A_{t}$ is defined by Equation (40).

(40) $$g_{u_{t}}^{l}\left(\exp\left(\xi \right)\right)=L_{g}\left(A_{t}\xi \right)+O\left({∥\xi ∥}^{2}\right)$$

As the dynamic system model $X_{t}$ satisfies the affine group property, the left-invariant error dynamics are independent of the system’s state. Moreover, the left-invariant error satisfies the log-linear theorem and its log [ $\xi \in R^{\mathrm{dimg}}$ ] satisfies the linear system as shown in Equation (30).

(41) $$\frac{d}{dt}\xi_{t}=A_{t}\xi_{t}+w_{t}$$

where $A_{t}$ is the linear system error coefficient. Moreover, $A_{t}$ can be represented by the Lie group as a representation of the state transition matrix of the discrete-time linear dynamic system Φ. As the IEKF adopts the Kalman filter properties, there are prediction and update stages. In the prediction stage, the state transition matrix is used in calculating the covariance matrix P using the Riccati equation as follows:

(42) $$P_{n}=\Phi P_{n-1}\Phi^{T}+{\hat{Q}}_{n-1}$$

where $P_{n}$ is the estimation covariance of state estimation uncertainty in matrix form, and Φ is the state transition matrix that is represented by the exponential map of the adjoint operator $A_{t}$ as described in Equation (43).

(43) $$\Phi =\exp_{m}\left(A_{t}\Delta t\right)$$

moreover, $A_{t}$ is obtained from the linearization of the left-invariant error dynamics ( $g_{u_{t}}^{l}$) by using first-order approximation as follows:

(44) $$\eta_{t}^{l}=\exp\left(\xi_{t}\right)\approx I_{d}+L_{g}\left(\xi_{t}\right)$$

where $\eta_{t}^{l}$ is derived from the log-linear property theorem as a result of the autonomous error dynamics theorem that defines the system as an affine group if its dynamics satisfy for all time epochs and t>0 [23,46].

(45) $$\frac{d\eta_{t}^{l}}{dt}=g_{u_{t}}^{l}\left(\eta_{t}^{l}\right)$$

where $g_{u_{t}}^{l}\left(\eta_{t}^{l}\right)$ is the Lie group of the left-invariant error dynamics $\xi_{t}$ and calculated as follows:

(46) $$\begin{array}{cc} \; & g_{u_{t}}^{l}\left(\eta_{t}^{l}\right)=g_{u_{t}}^{l}\left(I_{d}+L_{g}\left(\xi_{t}\right)\right)\\ \; & \left.=f_{u_{t}}\left(I_{d}+L_{g}\xi_{t}\right)\right)-f_{u_{t}}\left(I_{d}\right)\left(I_{d}+L_{g}\left(\xi_{t}\right)\right)\\ \; & =\left[\begin{array}{ccc} \xi_{t}^{v_{t}} & \left(I+{\left(\xi_{t}^{C_{t}}\right)}^{\wedge }\right){\tilde{f}}_{t}+g & \left(I+{\left(\xi_{t}^{C_{t}}\right)}^{\wedge }\right){\tilde{\omega }}_{t}^{\wedge }\\ 0 & 0 & 0_{1\times 3}\\ 0 & 0 & 0_{1\times 3} \end{array}\right]\\ \; & -\left[\begin{array}{ccc} 0_{3,1} & {\tilde{f}}_{t}+g & {\tilde{\omega }}_{t}^{\wedge }\\ 0 & 0 & 0_{1\times 3}\\ 0 & 0 & 0_{1\times 3} \end{array}\right]\left[\begin{array}{ccc} \xi_{t}^{p_{t}} & \xi_{t}^{v_{t}} & I+{\left(\xi_{t}^{C_{t}}\right)}^{\wedge }\\ 0 & 1 & 0_{1\times 3}\\ 1 & 0 & 0_{1\times 3} \end{array}\right]\\ \; & =\left[\begin{array}{ccc} G_{11} & G_{12} & G_{13}\\ 0 & 0 & 0_{1\times 3}\\ 0 & 0 & 0_{1\times 3} \end{array}\right] \end{array}$$

where I is the identity matrix and its dimension is 3×3, $\xi_{t}$ is the vector map of the navigation element (position, velocity, or rotation) of the Lie group, ${\left(\;\cdot \;\right)}^{\wedge }$ denotes a 3×3 skew-symmetric matrix, and *g* is the the modeled gravity as in Equation (9).

(47) $$\begin{array}{cc} G_{11} & =\xi_{t}^{v_{t}}-{\tilde{\omega }}_{t}^{\wedge }\xi_{t}^{p_{t}}\\ G_{12} & ={\left(\xi_{t}^{C_{t}}\right)}^{\wedge }{\tilde{f}}_{t}-{\tilde{\omega }}_{t}^{\wedge }\xi_{t}^{v_{t}}\\ G_{13} & ={\left(\xi_{t}^{C_{t}}\right)}^{\wedge }{\tilde{\omega }}_{t}^{\wedge }-{\tilde{\omega }}_{t}^{\wedge }{\left(\xi_{t}^{C_{t}}\right)}^{\wedge }={\left(-{\tilde{\omega }}_{t}^{\wedge }\xi_{t}^{C_{t}}\right)}^{\wedge } \end{array}$$

To simplify the previous equations, then $g_{u_{t}}^{l}\left(\eta_{t}^{l}\right)$ can be represented as shown in Equation (48).

(48) $$\begin{array}{cc} g_{u_{t}}^{l}\left(\eta_{t}^{l}\right) & =L_{g}\left(\left[\begin{array}{c} -{\tilde{\omega }}_{t}^{\wedge }\xi_{t}^{C}\\ -{\tilde{f}}_{t}^{\wedge }\xi_{t}^{C}-{\tilde{\omega }}_{t}^{\wedge }\xi_{t}^{v}\\ \xi_{t}^{v}-{\tilde{\omega }}_{t}^{\wedge }\xi_{t}^{p} \end{array}\right]\right)\\ \; & =L_{g}\left(\left[\begin{array}{ccc} -{\tilde{\omega }}_{t}^{\wedge } & 0_{3\times 3} & 0_{3\times 3}\\ -{\tilde{f}}_{t}^{\wedge } & -{\tilde{\omega }}_{t}^{\wedge } & 0_{3\times 3}\\ 0_{3\times 3} & I_{3\times 3} & -{\tilde{\omega }}_{t}^{\wedge } \end{array}\right]\left[\begin{array}{c} \xi_{t}^{C}\\ \xi_{t}^{v}\\ \xi_{t}^{p} \end{array}\right]\right) \end{array}$$

Accordingly, the covariance matrix, $P_{t}$, is computed using the Riccati equation as follows:

(49) $$\frac{d}{dt}P_{t}=A_{t}P_{t}+P_{t}\;A_{t}+{\hat{Q}}_{t}$$

where

(50) $$A_{t}=\left[\begin{array}{ccc} -{\tilde{\omega }}_{t}^{\wedge } & 0_{3\times 3} & 0_{3\times 3}\\ -{\tilde{f}}_{t}^{\wedge } & -{\tilde{\omega }}_{t}^{\wedge } & 0_{3\times 3}\\ 0_{3\times 3} & I_{3\times 3} & -{\tilde{\omega }}_{t}^{\wedge } \end{array}\right]$$

(51) $${\tilde{\omega }}_{t}=\omega_{t}+w_{t}^{g},{\tilde{f}}_{t}=f_{t}+w_{t}^{f}$$

where ${\left(\;\cdot \;\right)}^{\wedge }$ denotes a 3×3 skew-symmetric matrix and

(52) $${\hat{Q}}_{t}=\mathrm{Cov}\left[w_{t}\right]$$

Finally, the previous state system covariance is calculated as shown in Equation (53):

(53) $${\hat{Q}}_{n-1}\approx \Phi {\hat{Q}}_{t}\Phi^{T}\Delta t$$

From the previous derivation, it is clear that the linear system error coefficient, $A_{t}$, depends on the IMU measurements that are taken in the body frame and the covariance matrix P calculation depends on it.

This system model equation can be discretized and linearized as in Equation (54):

(54) $${\hat{X}}_{t_{n}}=\left[\begin{array}{ccc} {\hat{p}}_{t_{n}} & {\hat{v}}_{t_{n}} & {\hat{C}}_{t_{n}}\\ 0 & 1 & 0_{1\times 3}\\ 1 & 0 & 0_{1\times 3} \end{array}\right]$$

where $t_{n}$ is the discrete nature of the position, velocity, and attitude vectors, and the upper hat is used for the state’s estimations.

The measurement model is represented by Equation (55).

(55) $$Y_{t_{n}}={\hat{X}}_{t_{n}}b+\varepsilon_{t_{n}}$$

where $\varepsilon_{t_{n}}$ is the measurement error, and b is the measurement model’s design matrix and is represented as in Equation (56).

(56) $$b=\left[\begin{array}{c} 1\\ 0\\ 0\\ 0\\ 0 \end{array}\right]$$

The continuous dynamics can be discretized by assuming a zero-order hold on the inputs and performing quaternion integration from within a Δt time frame starting at $t_{n-1}$ and ending at $t_{n}$.

The discrete dynamics of each state element (position, velocity, and orientation) are represented by Equations (57)–(59), respectively.

The position states are derived as in Equation (57) using the previously known position and velocity in addition to the current state acceleration.

(57) $${\hat{p}}_{t_{n}}={\hat{p}}_{t_{n-1}}+{\hat{v}}_{t_{n-1}}\Delta t+\frac{1}{2}\left({\hat{C}}_{t_{n-1}}f_{t}+g\right)\Delta t^{2}$$

where ${\hat{p}}_{t_{n-1}}$ is the old position state, ${\hat{v}}_{t_{n-1}}$ is the old velocity, ${\hat{C}}_{t_{n-1}}$ is the quaternion transformation matrix from the body frame to the navigation frame using previous orientation angles, and $f_{t}$ is the current acceleration state.

(58) $${\hat{v}}_{t_{n}}={\hat{v}}_{t_{n-1}}+\left({\hat{C}}_{t_{n-1}}f_{t}+g\right)\Delta t$$

(59) $${\hat{C}}_{t_{n}}={\hat{C}}_{t_{n-1}}\exp\left(\omega_{t}\Delta t\right)$$

where Δt is the time update and exp(·) is the exponential map.

The linearized invariant error first-order approximation $\eta_{t_{n}}^{l}$ is represented by Equation (60) as follows [25,46,49]:

(60) $$\begin{array}{c} \eta_{t_{n}}^{l+}=\eta_{t_{n}}^{l}\exp\left(L_{t_{n}}\left({\left(\eta_{t_{n}}^{l}\right)}^{-1}b-b+{\hat{X}}_{t_{n}}^{-1}\varepsilon_{t_{n}}\right)\right) \end{array}$$

where $\eta_{t_{n}}^{l}$ is the old linearized invariant error first-order approximation, $L_{t_{n}}$ is the filter gain matrix, exp(·) is the exponential map, b is the measurement model’s design matrix, ${\hat{X}}_{t_{n}}^{-1}$ is the estimate of the system model states, and $\varepsilon_{t_{n}}$ is the measurement error.

The updated estimate of the system model states is shown in Equation (61).

(61) $$\begin{array}{c} {\hat{X}}_{t_{n}}^{+}={\hat{X}}_{t_{n}}\exp\left(L_{t_{n}}\left({\hat{X}}_{t_{n}}^{-1}Y_{t_{n}}-b\right)\right) \end{array}$$

The covariance update equation of the IEKF is shown in Equation (62).

(62) $$\begin{array}{c} P_{t_{n}}^{+}=\left(I-L_{t_{n}}H\right)P_{t_{n}}{\left(I-L_{t_{n}}H\right)}^{T}+L_{t_{n}}{\hat{N}}_{t_{n}}L_{t_{n}}^{T} \end{array}$$

where H is the measurement model linearized design matrix reduced form given as shown in Equation (63) [46]:

(63) $$\begin{array}{cc} H & =\left[\begin{array}{ccc} I_{3\times 3} & 0_{3\times 3} & 0_{3\times 3} \end{array}\right]\\ \; & =\left[\begin{array}{ccccccccc} 1 & 0 & 0 & 0 & 0 & 0 & 0 & 0 & 0\\ 0 & 1 & 0 & 0 & 0 & 0 & 0 & 0 & 0\\ 0 & 0 & 1 & 0 & 0 & 0 & 0 & 0 & 0 \end{array}\right] \end{array}$$

and

(64) $$\left\{\begin{array}{cc} {\hat{N}}_{t_{n}} & ={\hat{X}}_{t_{n}}^{-1}\;Cov\left[\varepsilon_{t_{n}}\right]{\hat{X}}_{t_{n}}^{-T}\\ L_{t_{n}} & =P_{t_{n}}H^{T}S^{-1}\\ S & =HP_{t_{n}}H^{T}+{\hat{N}}_{t_{n}} \end{array}\right.$$

A detailed derivation of the relation between *H* and *b* is explained using both autonomous error dynamics and log-linear property of the error theorems in [46].

#### 2.3.2. Measurement Model

The measurement model for the GPS readings $Y_{t_{n}}$ is represented as in Equation (55) [50].

Where

(65) $$Y_{t_{n}}=\left[\begin{array}{c} \varphi_{INS}-\varphi_{GPS}\\ \lambda_{INS}-\lambda_{GPS}\\ h_{INS}-h_{GPS}\\ 0\\ 1 \end{array}\right]$$

The expanded form of the measurement model is given as in Equation (66).

(66) $$\left[\begin{array}{c} \varphi_{INS}-\varphi_{GPS}\\ \lambda_{INS}-\lambda_{GPS}\\ h_{INS}-h_{GPS}\\ 0\\ 1 \end{array}\right]=\left[\begin{array}{ccccc} \delta \varphi & \delta V_{N} & q_{1}^{2}+q_{0}^{2}-q_{2}^{2}-q_{3}^{2} & 2(q_{1}q_{2}-q_{3}q_{0}) & 2(q_{2}q_{3}+q_{2}q_{0})\\ \delta \lambda & \delta V_{E} & 2(q_{1}q_{2}+q_{3}q_{0}) & q_{2}^{2}+q_{0}^{2}-q_{1}^{2}-q_{3}^{2} & 2(q_{2}q_{3}-q_{1}q_{0})\\ \delta h & \delta V_{D} & 2(q_{1}q_{3}-q_{2}q_{0}) & 2(q_{2}q_{3}+q_{1}q_{0}) & q_{3}^{2}+q_{0}^{2}-q_{1}^{2}-q_{2}^{2}\\ 0 & 1 & 0 & 0 & 0\\ 1 & 0 & 0 & 0 & 0 \end{array}\right]\left[\begin{array}{c} 1\\ 0\\ 0\\ 0\\ 0 \end{array}\right]+\varepsilon_{t_{n}}$$

where the measurements covariance matrix $R_{n}$ is given as in Equation (22).

(67) $$R_{n}=\left[\begin{array}{c} \sigma_{\varphi }^{2}\\ \sigma_{\lambda }^{2}\\ \sigma_{h}^{2}\\ 0\\ 0 \end{array}\right]$$

**Figure 2:**
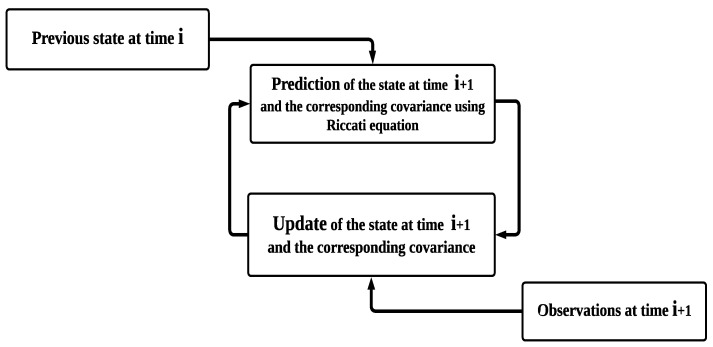
A simplified block diagram of the IEKF algorithm.

A simplified block diagram of the IEKF algorithm shown in Figure 2 indicates the process of the prediction and the obtained corresponding covariance matrix using the Riccati equation. Moreover, the update of the states and the updated covariance matrix is also mentioned.

### 2.4. INS-GPS Integration Using IEKF

**Figure 3:**
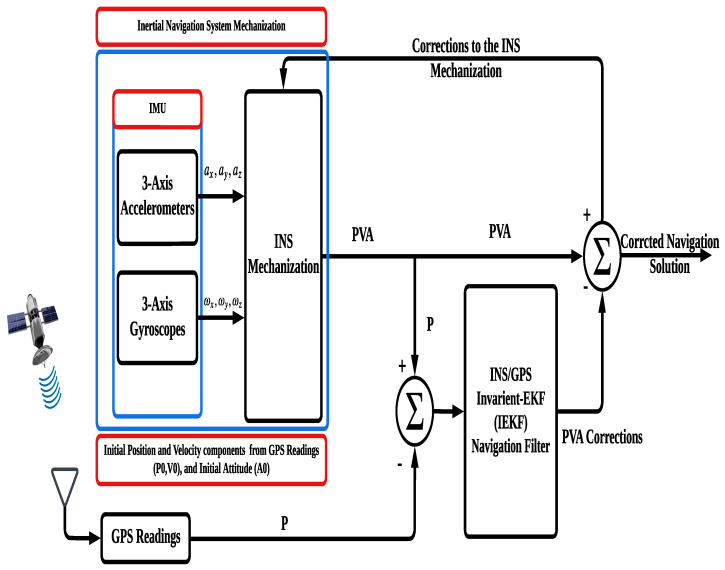
The INS/GPS-based IEKF integrated navigation system block diagram.

Despite the GPS’s long-term stability and the INS’s short-term stability, the fusion/ integration between the two systems provides a better solution than each of them separately. The integration scheme shown in Figure 3 is a loosely coupled framework. However, in this type of integration, a GPS solution is a main requirement for the filter to work properly. The INS system provides a continuous PVA solution with unbounded overtime drift [4]. Therefore, aiding information is demanded for error control and solution enhancement. The GPS provides position readings to update the INS.

Traditionally, the extended Kalman filter (EKF) is the utilized navigation filter [51]. However, the EKF in general is not an optimal estimator, used for nonlinear systems [8], as it will fail if the functions are highly nonlinear (1st-order approximation) because its state estimates depend on Jacobians, but invariant extended Kalman filter states are not the typical Jacobian linearization along a trajectory because the invariant error on the Lie group can be exactly recovered from its solution.

The main advantage of IEKF over EKF is that the state linearization and measurement models are independent of the current estimation of the state leading to state-independent Jacobians at any linearization point [27,28]. In this work, an IEKF works as a navigation filter. The algorithm provides accurate positioning information.

## 3. Experimental Setup

**Figure 4:**
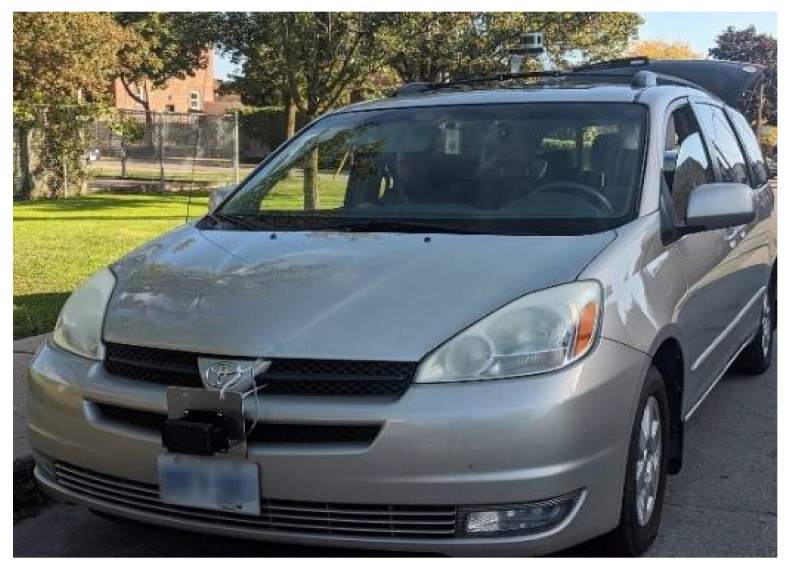
The land vehicle that is used in the experiment.

**Figure 5:**
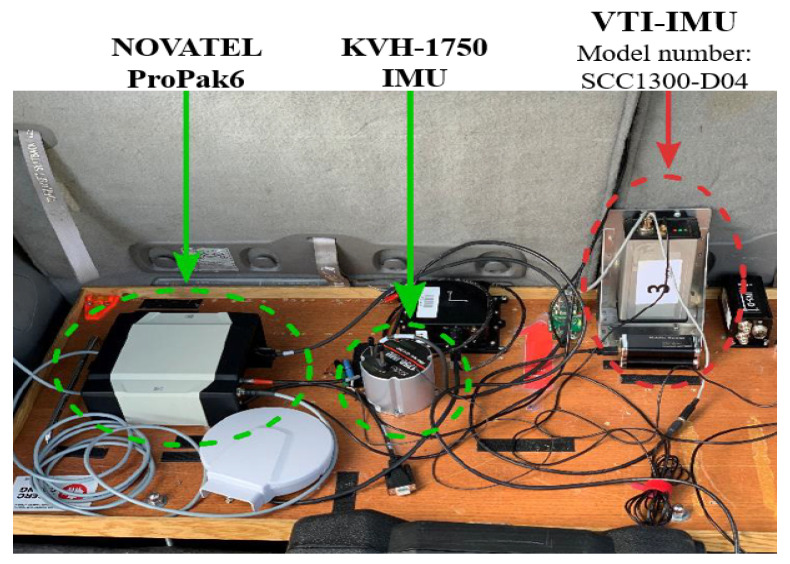
The utilized sensors/systems mounted on the internal testbed.

A real road trajectory was conducted to study the proposed method’s performance in the urban area of the city of Kingston, Ontario, Canada in 2020. The vehicle used in the conducted trajectory is shown in Figure 4 and three major units are used as shown in Figure 5. The reference is provided by a tightly coupled INS/GPS integration between the Novatel Propack6 GPS receiver and the KVH-1750 IMU. Those units’ specifications are described as follows [52]:

- NOVATEL ProPak6 is a GNSS receiver with a 2D-position accuracy of less than 1.5 m, a velocity accuracy of less than 0.03 m/s, and a time accuracy of 20 ns. For the purpose of this experiment, its data rate that can reach 100 Hz is adjusted to 1 Hz.
- KVH-1750 IMU’s gyroscopes have a bias instability of $\leq 0.05^{\circ }/$ hr, 1σ, a maximum of $\leq 0.1^{\circ }/\mathrm{hr},1\sigma$, with bias offset $\pm 2^{\circ }/\mathrm{hr}$, and angle random walk of $\leq 0.012^{\circ }/\sqrt{\mathrm{hr}}$ $\left(\leq 0.7^{\circ }/\mathrm{hr}/\sqrt{\mathrm{Hz}}\right)$. The IMU’s accelerometers have a bias instability of $\;0.01{\mathrm{mg}}^{-1\sigma }$, a scale factor linearity of 0.008, and a velocity random walk of $0.024\;\mathrm{mg}/\sqrt{\mathrm{Hz}}$. For the purpose of this experiment, its data rate that can reach 1000 Hz is adjusted to 100 Hz
- VTI-IMU (model number: SCC1300-D04) is a low-cost MEMS-grade IMU. It has a gyro bias drift of 1.5 deg/s, a scale factor linearity ≤2%, a velocity random walk of 0.86 deg/$\sqrt{\mathrm{Hz}}$, and an update rate of 20 Hz.

## 4. Results and Discussion

The IEKF filter is applied to a loosely coupled INS/GPS integration for AV navigation. The results show a significant improvement in the positioning information provided to the vehicle compared to the traditional EKF.

**Figure 6:**
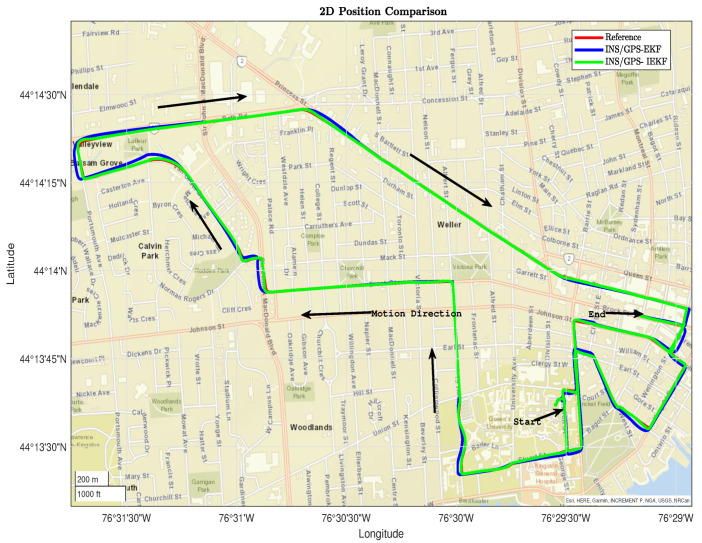
The conducted trajectory overlaid onto a digital map.

The proposed INS/GPS-IEKF integrated navigation system performed better during several dynamics, such as straight driving, turns, and consecutive turns at various speeds. Figure 6 shows the overall trajectory overlaid onto a digital map based on the reference from a tightly coupled INS/GPS integration between the Novatel Propack6 GPS receiver and the KVH-1750 IMU compared to the INS/GPS-based EKF and IEKF.

**Figure 7:**
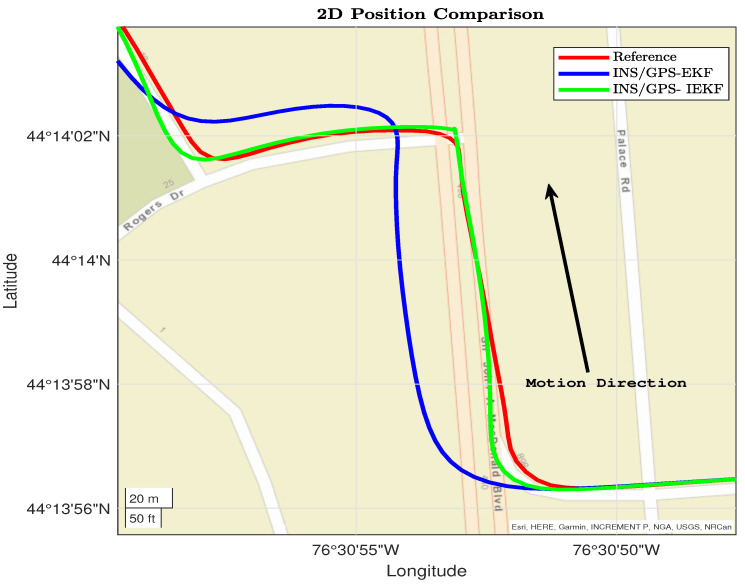
First dynamic section for reference, EKF, and IEKF.

Moreover, the performance of the two applied filters is tested using two different trajectory parts. The first part is shown in Figure 7 and contains several dynamics such as a right turn, straight driving, and left–right turns. In this part, the INS/GPS-based EKF diverges, unlike the INS/GPS-based IEKF, which converges with the reference.

**Figure 8:**
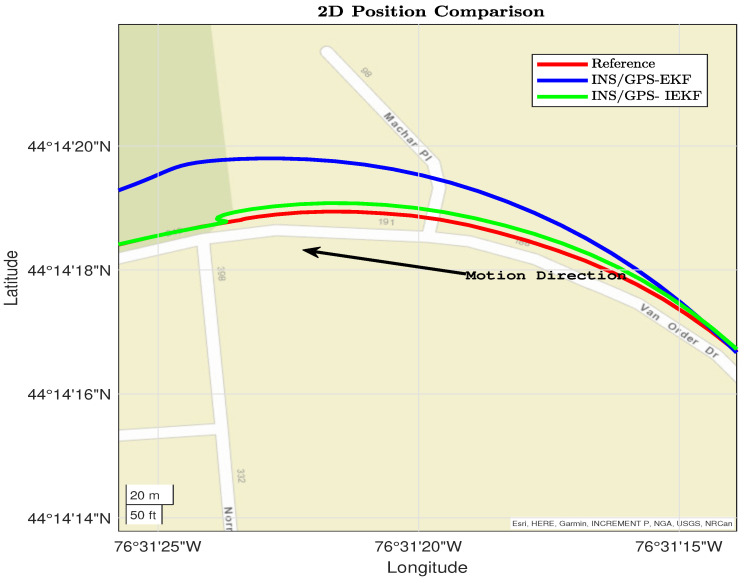
Second dynamic section for reference, EKF, and IEKF.

The second part of the trajectory shown in Figure 8 involves a slight left turn with a natural GPS outage. The proposed INS/GPS-based IEKF outperformed the traditional INS/GPS-based EKF. The proposed system provides a better position solution with a minimum drift compared to the INS/GPS-based EKF. Moreover, when the GPS readings became available, the proposed system converged directly, unlike the INS/GPS-based EKF system, which suffers from a convergence delay because of the drift error estimation.

**Figure 9:**
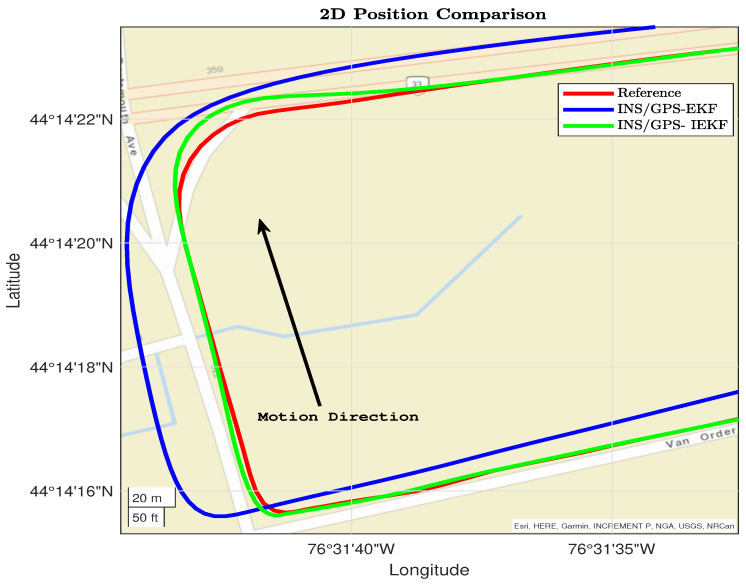
Third dynamic section for Reference, EKF, and IEKF.

Furthermore, Figure 9 shows two dynamics, straight driving between two right turns. The proposed INS/GPS-based EKF still diverges unlike the IEKF that keeps on its convergence with the reference.

**Figure 10:**
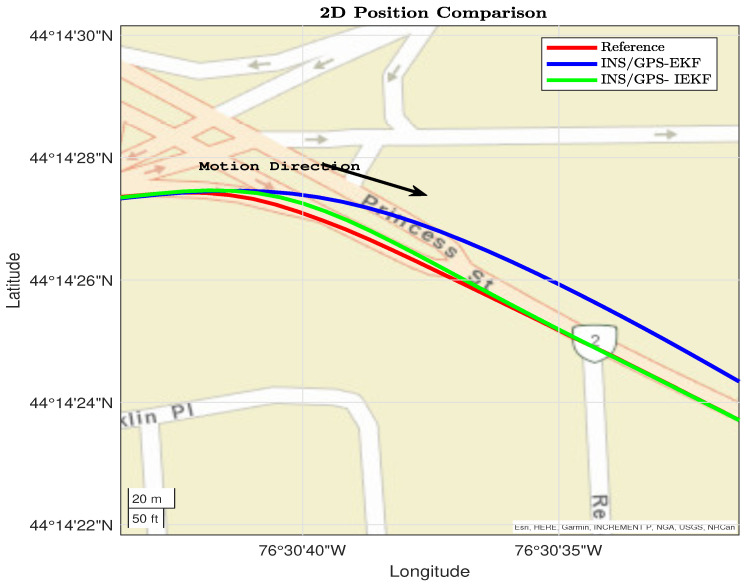
Fourth dynamic section for reference, EKF, and IEKF.

Figure 10 involves a slight turn; moreover, the performance of the proposed INS/GPS-based IEKF is better than the performance of the proposed INS/GPS-based EKF.

From Figure 7, Figure 8, Figure 9 and Figure 10 the performance of the proposed INS/GPS-based IEKF and the INS/GPS-based EKF have close position estimation during straight driving. On the other hand, during the dynamics the proposed integrated system provides a much more accurate position compared to the traditional system. This significant improvement is due to the capability of the proposed INS/GPS-based IEKF in estimating the IMU associated nonlinear error components compared to the linearized EKF.

Finally, the estimated 2D-position RMS error of the proposed INS/GPS-based IEKF compared to its correspondence from the INS/GPS-based EKF over the whole trajectory is shown in Figure 10.

**Figure 11:**
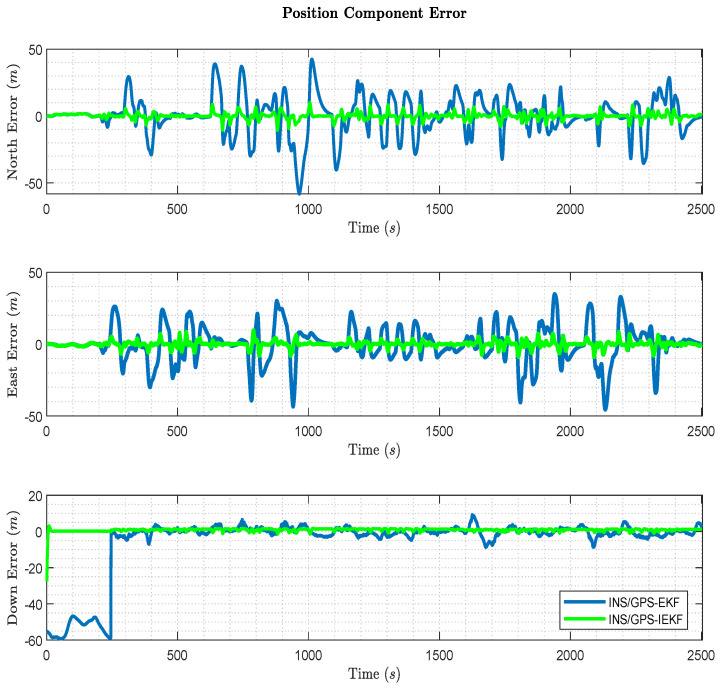
Position component error comparison in the NED frame.

Moreover, a comparison between the position component errors in the north, east, and down frames between the traditional INS/GPS-based EKF and the proposed INS/GPS-based IEKF is shown in Figure 11. The results show that the output north and east position component errors when applying the proposed method are less than the traditional INS/GPS-based EKF over the whole trajectory. This leads to a better 2D-position estimation when using the proposed method. Furthermore, the altitude error is significantly decreased when using the proposed method which results in a better 3D-position estimation. However, the EKF altitude results in a significant error during the first stationary 3 min due to the start location. This location suffered from high vertical dilution of precision (VDOP) during the experiment. Therefore, a divergence occurred until the vehicle started to move as the results imply. Moreover, the EKF state correction depends on a time-based GPS update and uses the last reading in the measurement model which leads to a solution drift if the updates are incorrect or blocked. On contrary, during the same period of time the IEKF altitude results in a better estimation due to the right estimation of the INS errors. Generally, the proposed method provides a better position estimation than the traditional one.

For further analysis, Table 1 shows both mean and standard deviation (STD) of each system’s position component error. The results confirm the superiority of the proposed system as both mean and STD of the north, east and down (NED) position component error are minimum when applying the proposed INS/GPS-based IEKF system.

**Figure 12:**
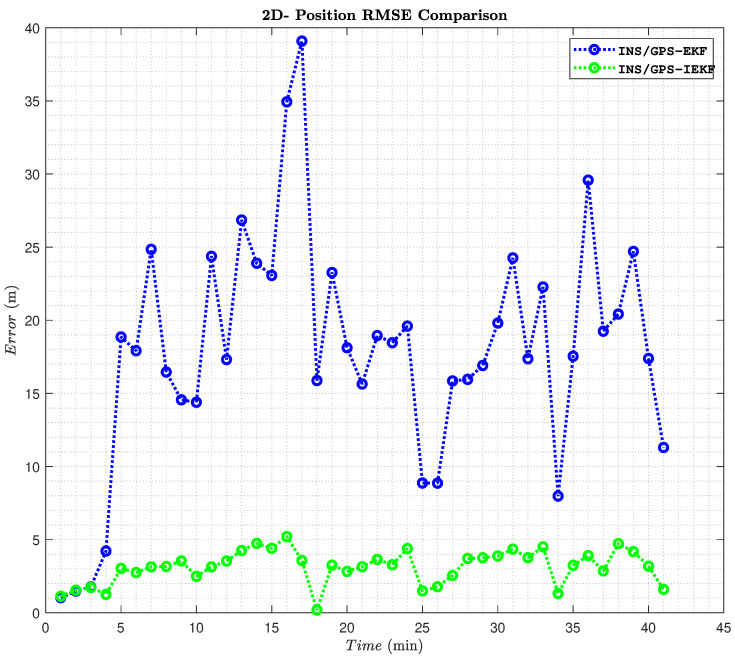
A 2D-position RMS error comparison per minute over the whole trajectory.

Furthermore, the whole trajectory minute by minute RMS error comparison between the two systems is shown in Figure 12. In detail, the overall trajectory takes about 41 min involving a stationary three minutes in the beginning. During the first three minutes there were INS alignment processes and the GPS readings had a high DOP error due to the location surroundings. This stationary period is used for checking the performance of the two systems in a no-motion state. Afterward, when the vehicle started to move a significant change in performance appears.

Clearly, at minute number 5 the proposed INS/GPS-based EKF diverges due to high speed unlike the performance of the proposed INS/GPS-based IEKF that converges with the reference. Moreover, the max RMS error occurred at the 17th minute for both systems. However, during this exact minute, the average speed was almost 16.5 m/s and the travel distance was almost 1 km. In particular, the proposed INS/GPS-based IEKF system with a 2D-position RMS error reached 5.5 m, while the INS/GPS-based EKF reached 39 m within this particular period.

Generally, the performance of the proposed system during the whole trajectory produced a significant reduction in the 2D-position RMS error which provides a great enhancement of the position information provided to the vehicle. The average 2D-position RMS error was reduced from 19.3 m to 3.3 m when applying the proposed INS/GPS-based IEKF algorithm with an enhancement percentage of 82.9%. More details about the results are illustrated in Table 2 and Table 3.

Table 2 shows the position max errors. The tabulated results show a significant enhancement in the 3D position when using the proposed INS/GPS-based IEKF system due to the superiority in the altitude estimation unlike the traditional system INS/GPS-based EKF system. Moreover, from Table 2 the 2D-position max error reduced from 73.9 m to 14.2 m with 80.78% enhancement and the 3D-position max error reduced from 94.7 m to 30.9 m with 67.37% enhancement.

The results presented in Table 3 provide information on the position RMS errors of each position component (N, E, and D) as well as the 2D and 3D position RMSE for both systems. The proposed INS/GPS-based IEKF system exhibited a significant improvement for all position components, with a particularly noticeable enhancement in the 3D position due to its superior altitude estimation when compared to the traditional INS/GPS-based EKF system. Specifically, the 3D-position RMS error was reduced from 25.7 m to 3.5 m, representing an 86.38% improvement. Similarly, the 2D-position RMS error was reduced from 19.4 m to 3.3 m, indicating an 82.98% improvement.

**Figure 13:**
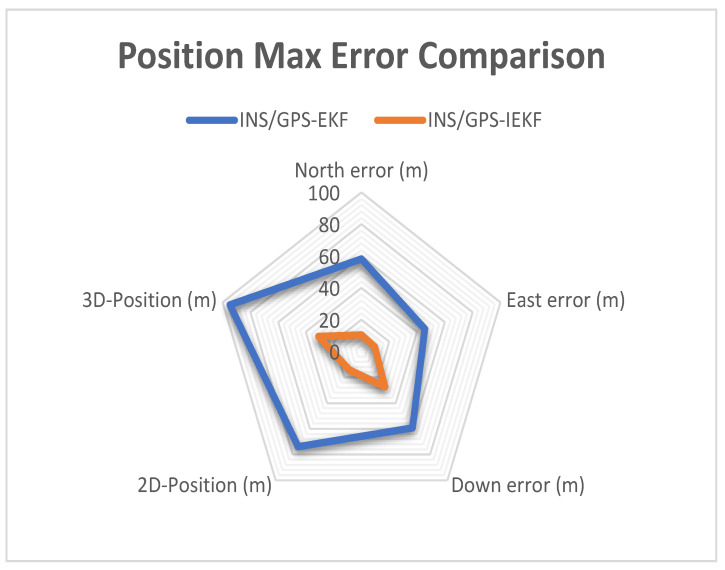
Radar map for position max error comparison.

**Figure 14:**
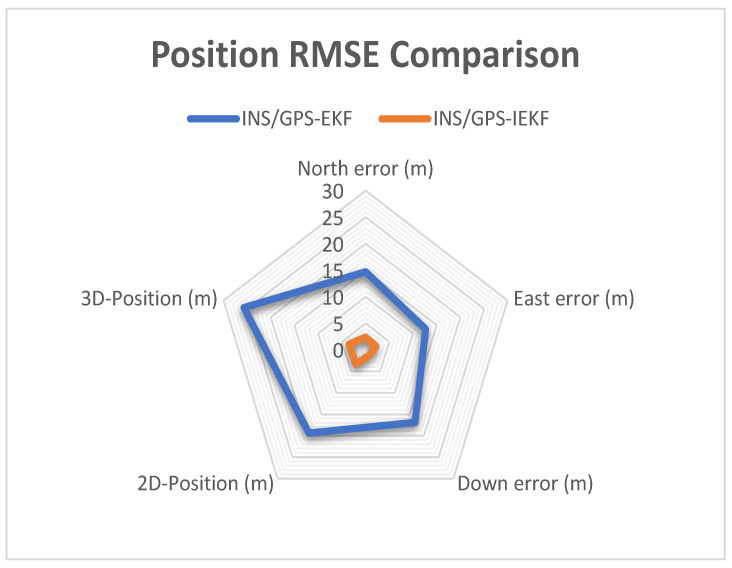
Radar map for position RMSE comparison.

To compare the performance of the proposed INS/GPS-based IEKF system and the traditional INS/GPS-based EKF system in a more intuitive way, two radar maps depicting the maximum error and RMSE of position parameters are presented in Figure 13 and Figure 14. These figures reveal that the proposed system exhibits a smaller error area as compared to the traditional system. In conclusion, the results demonstrate that the proposed INS/GPS integrated navigation system utilizing IEKF offers superior error estimation and provides more accurate position information than the EKF-based system.

## 5. Conclusions

In this paper, we proposed an IEKF filter for a loosely coupled INS/GPS integration scheme to improve the navigation of autonomous vehicles. The proposed method resulted in a significant improvement in the accuracy of the positioning information provided to the vehicle. We presented a detailed explanation of the proposed integrated navigation system and tested it over a real road trajectory. Our results demonstrated that the proposed INS/GPS-based IEKF system outperformed the traditional INS/GPS-based EKF system during various vehicle dynamics such as straight driving, turns, and consecutive turns at different speeds. Specifically, the 2D-position RMSE was reduced from 19.4 m to 3.3 m with an 82.98% improvement, and the 3D-position RMSE was reduced from 25.7 m to 3.5 m with an 86.38% improvement. Furthermore, the 2D-position max error was reduced from 73.9 m to 14.2 m with an 80.78% improvement, and the 3D-position max error was reduced from 94.7 m to 30.9 m with a 67.37% improvement compared to the traditional system.

For future studies, we plan to assess the proposed approach under various GPS outage scenarios and expand the updates to include both velocity and IMU sensor measurements. Additionally, we will conduct a performance comparison study of the four systems: INS/GPS/EKF-based Euler, INS/GPS/IEKF-based Euler, INS/GPS/EKF-based quaternion, and INS/GPS/IEKF-based quaternion.

## Figures and Tables

**Table 1:** Statistical analysis of the NED position component error.

	Mean Error		STD	
	INS/GPS-EKF	INS/GPS-IEKF	INS/GPS-EKF	INS/GPS-IEKF
**North**	10.35 m	1.56 m	14.73 m	2.45 m
**East**	8.87 m	1.36 m	12.66 m	2.21 m
**Down**	6.85 m	0.97 m	16.01 m	0.93 m

**Table 2:** Position max error comparison.

	INS/GPS-EKF	INS/GPS-IEKF
**North**	58.2 m	10.5 m
**East**	45.6 m	9.5 m
**Down**	59.3 m	27.4 m
**2D Position**	73.9 m	14.2 m
**3D Position**	94.7 m	30.9 m

**Table 3:** Position RMSE comparison.

	INS/GPS-EKF	INS/GPS-IEKF
**North**	14.72 m	2.45 m
**East**	12.7 m	2.2 m
**Down**	16.9 m	1.3 m
**2D Position**	19.4 m	3.3 m
**3D Position**	25.7 m	3.5 m

## Data Availability

Not applicable.
